# Development and Validation of the Evaluating Attitudes, Training and Skills in Dysphagia Care (EATS) Questionnaire Among Nursing Home Nurses in Singapore

**DOI:** 10.3390/nursrep15110405

**Published:** 2025-11-17

**Authors:** Laurence Lean Chin Tan, Yujun Lim, Gerlie Contreras Magpantay, James Alvin Low

**Affiliations:** 1 Department of Geriatric Medicine and Palliative Medicine, Khoo Teck Puat Hospital, Singapore 768828, Singapore; low.james.yh@nhghealth.com.sg; 2 Yong Loo Lin School of Medicine, National University of Singapore, Singapore 117597, Singapore; 3 Independent Researcher, Singapore 759150, Singapore; yj.lim026@gmail.com; 4 Community Health, NHG Population Health, Singapore 308205, Singapore; gerlie.c.magpantay@nhghealth.com.sg; 5 Geriatric Education and Research Institute (GERI), Singapore 768024, Singapore

**Keywords:** dysphagia, nursing home, questionnaire validation, nursing competency, nurses’ knowledge, nurses’ attitudes, long-term care

## Abstract

**Background**: Dysphagia is prevalent among nursing-home residents and contributes to complications such as aspiration pneumonia, malnutrition, and diminished quality of life. Nurses’ knowledge and attitudes strongly influence care quality, yet few validated tools exist to assess these domains in long-term care. **Objective:** This study aimed to develop and validate the Evaluating Attitudes, Training and Skills in Dysphagia Care (EATS) Questionnaire for nursing home nurses in Singapore. **Methods**: A cross-sectional study involving 111 nurses from three nursing homes was conducted. The EATS questionnaire was adapted from a hospice-based tool, refined through experts’ and users’ feedback, and subjected to psychometric testing. Analyses included item difficulty and discrimination for the knowledge component, exploratory factor analysis for the attitude component, and internal-consistency reliability. Construct validity was examined by comparing knowledge and attitude scores across nursing seniority and experience. **Results**: The final questionnaire comprised 22 knowledge and 18 attitude items that loaded onto three factors—Barriers to Dysphagia Care, Patients’ Preferences and Nurses’ Confidence, and Personal Choice. The attitude scale showed moderate internal consistency, and the knowledge items demonstrated acceptable performance for discrete factual content. Senior nurses scored higher in knowledge, confirming construct validity. The tool revealed persistent misconceptions and gaps in recognising subtle clinical signs of dysphagia. **Conclusions**: The EATS Questionnaire is a valid and pragmatic instrument for assessing dysphagia-related knowledge and attitudes among nursing home nurses. It provides actionable insights for designing targeted education and improving resident safety in long-term care settings.

## 1. Introduction

Dysphagia, defined as difficulty in swallowing, is a common and serious condition among nursing home residents and patients receiving palliative care [1]. It is associated with significant adverse outcomes, including aspiration pneumonia, malnutrition, dehydration, social withdrawal, and diminished quality of life [2,3]. Despite these profound implications, dysphagia frequently remains under-recognised and inconsistently managed within long-term care settings [4], compromising care quality and patient safety [5].

Nurses play a central role in feeding assistance, oral hygiene, and the early recognition of swallowing difficulties, as they are primarily responsible for implementing dietary modifications and monitoring residents’ swallowing safety on a daily basis [6]. Their knowledge of dysphagia-related risks and attitudes towards care interventions are critical determinants of effective management of dysphagia and resident outcomes [7].

Despite their crucial role, studies consistently show that nurses receive limited or inconsistent training in dysphagia management, leading to variability in practice, inappropriate feeding strategies, and missed opportunities for early intervention [8]. Inadequate knowledge and negative attitudes toward feeding care can have serious consequences for residents’ safety, nutrition, and quality of life [9]. Yet, there remains a paucity of validated instruments specifically designed to assess both the knowledge and attitudes of nursing-home nurses. Establishing a psychometrically sound assessment tool is therefore essential for identifying learning needs, evaluating the impact of training programmes, and strengthening dysphagia-care standards across long-term-care settings.

Several instruments have been developed to assess dysphagia-related knowledge and attitudes, but they were designed for different populations and settings. The Caregiver Mealtime and Dysphagia Questionnaire (CMDQ) [10] evaluates caregivers’ perceptions of mealtime difficulties and burden, rather than professional knowledge. Recent studies have investigated knowledge, attitudes, and practices among healthcare professionals but were focused on hospital healthcare professionals [11] or focused on dysphagia assessment rather than management [7]. Speyer et al. also surveyed multidisciplinary professionals on dysphagia-care knowledge and training needs [12]. However, this tool was used for hospital or rehabilitation settings and have not been validated for nursing home nurses. In long-term care, nurses manage daily feeding, oral care, and aspiration prevention. These tasks require both sound knowledge and the right attitudes toward dysphagia care.

To address this gap, the Evaluating Attitudes, Training and Skills in Dysphagia Care (EATS) Questionnaire was developed. This instrument uniquely integrates measures of factual knowledge—covering the complications, clinical signs, diet modifications, safe feeding practices, and enteral feeding—with assessment of attitudinal domains such as confidence level, perceived barriers, and personal perspectives toward dysphagia care. This study aims to validate the EATS Questionnaire among nursing home nurses in Singapore, rigorously examining item refinement, internal reliability, and construct validity to provide a robust tool for research, education, and quality improvement.

This study was conducted in Singapore, where nursing home care is delivered by a culturally and linguistically diverse workforce caring for an ageing population with a high burden of dysphagia-related complications. The inclusion of Singapore in the study title reflects this distinctive practice environment and supports contextual relevance for similar multicultural long-term-care systems.

## 2. Materials and Methods

Study Design and Setting. A cross-sectional validation study was conducted among three nursing homes in Singapore to evaluate the psychometric properties of the Evaluating Attitudes, Training and Skills in Dysphagia Care (EATS) Questionnaire. The study focused on item refinement, reliability, and construct validity of the instrument designed to assess knowledge and attitudes toward dysphagia care.

Participants. A convenience sample of 111 nursing staff of various designations working in nursing homes across Singapore participated in the study. Eligibility criteria were: direct care experience with residents who have swallowing difficulties and willingness to participate voluntarily and anonymously. No exclusion criteria based on years of experience or position were applied, to capture a representative sample of nursing home nurses.

Instrument Development and Preliminary Validation. The original pool of items for the EATS Questionnaire was developed for a study in an inpatient hospice based on expert consensus within a multidisciplinary workgroup consisting of a speech therapist, two senior nurses, and a palliative care physician. This item pool was independently reviewed by an external speech therapist and physician, and pre-tested for clarity with five doctors. The initial developmental phase and initial content validation were previously published [13].

For the present study, the setting was shifted from the hospice context to nursing homes, and wording changes were made to ensure the items were understandable and relevant to nursing home nurses. Content validity [14] of the revised item pool was further assessed by four practising speech therapists, who reviewed each item for clarity, comprehensiveness, and clinical relevance. Face validity [15] was then tested among 36 nursing home nurses to confirm item clarity and usability in the intended practice setting with no substantial changes needed. Correlating questionnaire responses with observed feeding practices or standardised simulation scenarios was not undertaken in this phase, as the present study focused on initial instrument validation.

Instrument Description. The refined questionnaire comprised two sections: a knowledge section with initially 34 items scored as true/false/uncertain responses, covering five domains (complications, signs, modified diets and fluids, safe feeding practices, and enteral feeding); and an attitude section with 23 items scored on a five-point Likert scale, designed to capture attitudes related to importance of aspiration prevention, patients’ preferences, perceived barriers, and personal choices.

Data Collection. Paper questionnaires were administered to 111 nurses across three nursing homes in Singapore. Demographics included age, nationality, designation, years in current occupation, and years in palliative care.

Psychometric Analysis. For knowledge, we computed item difficulty (% correct) and corrected item–total correlations [16]. Removal criteria were: item–total correlation < 0.20, extreme difficulty (≥95% or ≤10% correct), or limited clinical relevance [17]. Internal consistency was estimated with KR-20 [18].

For attitudes, we performed exploratory factor analysis (EFA) [19] (principal axis factoring, varimax rotation). Suitability was confirmed (KMO = 0.735; Bartlett’s χ^2^ = 1210.7, df = 253, *p* < 0.001). Factor retention combined scree plot and parallel analysis [20]. Items with loadings <0.40 or substantial cross-loadings were removed [21]. Internal consistency of the retained attitude factors was assessed with Cronbach’s alpha coefficients [22].

Final Scale Composition. Following iterative item reduction based on statistical criteria and clinical judgment, the final validated EATS questionnaire consisted of 22 knowledge items and 18 attitude items grouped into three factors: Barriers to Dysphagia Care, Patients’ Preferences and Nurses’ Confidence, and Personal Choice.

Ethics Statement and Data Protection. This study involved anonymous survey data from nursing home nurses. No identifiable personal information was collected, and participation was entirely voluntary. In line with institutional guidelines (DSRB-271023) drawn from national guidelines, formal ethics approval was not required for the analysis of anonymised, non-identifiable data. Information on nationality and ethnicity was collected solely to characterise the multicultural nursing workforce in Singapore’s long-term-care sector, which comprises nurses from diverse countries of origin. No subgroup analysis or reporting was performed, and all data were anonymised and aggregated to prevent identification of individuals or specific facilities. Completed questionnaires were stored in electronic datasets on password-protected institutional drives accessible only by the study team.

## 3. Results

A total of 111 nurses from three nursing homes completed the questionnaire. The mean age was 31.4 years (SD = 6.5), with an average of 7.3 years (SD = 10.0) of occupational experience and 1.8 years (SD = 3.4) in palliative care. Almost half of the respondents were nursing aides, and the majority were of Filipino nationality (Table 1).

The initial 29-item knowledge scale demonstrated item difficulty ranging from 10% to 99% correct. Several items showed ceiling effects, such as aspiration pneumonia (97% correct), reduced oral intake (98%), and monitoring during feeding (99%). Conversely, some items were highly challenging, including drooling (13%) and retained food in the mouth (10%). Item–total correlations ranged from −0.17 to 0.49, with a cut-off of ≥0.20 applied for acceptable discrimination (Table S1, Supplementary Materials).

Nine items were removed after psychometric testing due to poor correlations, extreme difficulty indices, or limited clinical or conceptual relevance. The final scale contained 22 knowledge items, encompassing complications, signs, modified diets and fluids, safe feeding practices, and enteral feeding.

The attitude section consisted of 23 items initially. EFA confirmed suitability of the dataset (KMO = 0.735; Bartlett’s χ^2^ = 1210.7, df = 253, *p* < 0.001). Scree plot inspection suggested either three or four factors, while parallel analysis supported a three-factor solution.

Varimax rotation yielded a clear and interpretable factor structure (Table S2, Supplementary Materials). The first factor, Barriers to Dysphagia Care, reflected perceptions of burden and practical challenges, such as the time taken to prepare thickened fluids, doubts about diet effectiveness, and the stress of feeding. The second factor, Patients’ Preferences and Nurses’ Confidence, encompassed attitudes regarding patients’ acceptance of diet modifications and nurses’ confidence in recognising aspiration. The third factor, Personal Choice, captured nurses’ own hypothetical choices in the context of terminal illness, including willingness to accept diet modifications or tube feeding.

**Figure 1:**
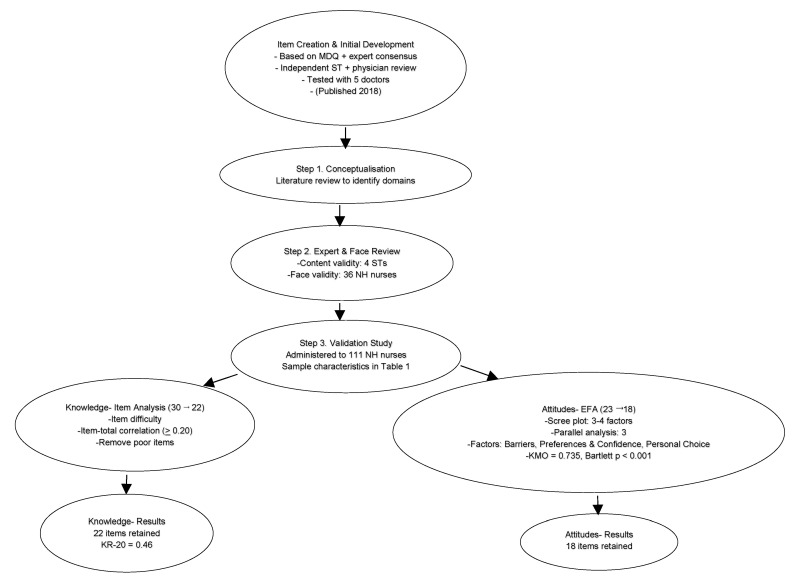
Development and validation pathway for the EATS Questionnaire. Steps included item generation (adapted from a palliative-care context to the nursing-home setting), expert content review (speech therapists), face validity testing (36 nursing-home nurses), main administration (*N* = 111), item analysis, and EFA-based reduction to the final scale (22 knowledge, 18 attitude). Developmental process was previously described in Tan L et al. (2018). Copyright 2018 The Hospice and Palliative Nurses Association.

Five items (Q4, Q5, Q6, Q18, Q19) were removed. While some had factor loadings <0.40, others were excluded because they were negatively worded or conceptually functioned more as knowledge items rather than attitudes. For example, Q4 and Q5 (‘preventing aspiration will not improve quality of life/symptoms’) loaded at ~0.50 but were deemed unsuitable as attitudinal items. Similarly, Q6 (‘thickened fluids improve quality of life’) overlapped with knowledge content. Q18 and Q19 showed weak loadings (<0.40) and were also negatively worded, reducing clarity. Figure 1 summarises development, expert/face review, and item reduction from the original pool to the final validated scale (22 knowledge, 18 attitude).

### 3.1. Reliability

The internal consistency of the knowledge and attitude scales was assessed. For the knowledge scale, one item (Q28: “Patients must be checked for food in the mouth after feeding”) showed zero variance, with all participants answering it correctly. This item was excluded from the reliability analysis. The remaining 21-item knowledge scale yielded a Kuder–Richardson 20 (KR-20) coefficient of 0.39, reflecting low internal consistency. For the attitude scale (18 Likert-type items), Cronbach’s α was 0.64, indicating moderate internal consistency.

### 3.2. Knowledge and Attitude Scores

The mean knowledge and attitude scores by designation are presented in Table 2. Knowledge scores differed significantly between designations (Kruskal–Wallis H(6) = 19.41, *p* = 0.004), with senior staff nurses and directors of nursing achieving the highest scores. In contrast, attitude scores did not differ significantly across designations (H(6) = 6.72, *p* = 0.35).

### 3.3. Correlations with Experience

The relationships between knowledge and attitude scores and years of experience are shown in Table 3. Neither years of nursing experience nor years of palliative care experience were significantly correlated with knowledge or attitude scores. Specifically, knowledge was weakly and non-significantly correlated with years in nursing (ρ = 0.04, *p* = 0.65) and years in palliative care (ρ = 0.13, *p* = 0.16). Attitudes were not correlated with years in nursing (ρ = −0.04, *p* = 0.64) or years in palliative care (ρ = 0.04, *p* = 0.71).

## 4. Discussion

This study developed and validated the Evaluating Attitudes, Training and Skills in Dysphagia Care (EATS) Questionnaire, the first tool to measure both dysphagia knowledge and attitudes among nursing home nurses in Singapore. The final questionnaire contained 22 knowledge items and 18 attitude items (Appendix A).

The relatively low internal consistency [23] (KR-20 = 0.39) observed in the knowledge scale reflects the heterogeneity of factual items rather than a measurement weakness. Knowledge tests in patient-safety contexts, unlike scales measuring a single construct [18] are intentionally broad and cover distinct, non-overlapping aspects of care (e.g., complications, safe feeding, enteral feeding). Such tests aim to ensure a minimum standard of safe practice, identifying those who may lack essential knowledge rather than ranking proficiency. Hence, a lower internal consistency is expected and acceptable for formative safety assessments even though it lowers statistical homogeneity [24].

Item selection in the EATS knowledge scale therefore, balanced psychometric performance with clinical utility. Items with weaker statistical properties were retained when they represented core safety principles or common misconceptions with direct implications for patient harm.

This pragmatic approach aligns with the intended purpose of the EATS questionnaire, which is to serve as a clinical and educational tool for workforce training rather than a high-stakes summative test. By preserving items that highlight misconceptions or safety-critical practices, the instrument prioritises its role in improving care quality and identifying training needs within the nursing-home sector, where ensuring that every nurse is ‘safe enough’ is more important than distinguishing the high performers.

The attitude scale showed clearer psychometric performance. Exploratory factor analysis (EFA) confirmed sampling adequacy (KMO = 0.735; Bartlett’s χ^2^ = 1210.7, *p* < 0.001) and supported a three-factor model. The final 18-item scale had a Cronbach’s alpha of 0.64, which is acceptable for a new scale that spans different domains [25]. The three factors were Barriers to Dysphagia Care, Patients’ Preferences and Nurses’ Confidence, and Personal Choice. These domains reflect real challenges in nursing homes, including workload, balancing safety with quality of life, and the influence of personal values on professional decisions.

The results highlight specific knowledge and attitude patterns about dysphagia. Similar to other studies, almost all respondents recognised aspiration pneumonia and reduced oral intake as consequences of dysphagia, showing that basic knowledge is well established [26]. In contrast, very few recognised drooling or retained food as warning signs, and many held misconceptions about enteral feeding [27]. These findings identify high-priority areas for education. Attitudes did not differ by designation or years of experience, suggesting that barriers and value judgments are common across all staff and are influenced more by shared experiences and workplace context than by seniority, consistent with studies showing that healthcare professionals’ attitudes are shaped more by organizational culture and common challenges than by hierarchical status or tenure [28].

The EATS Questionnaire has clear clinical and educational relevance. Knowledge results can be used to target teaching on under-recognised signs and to correct misconceptions that may compromise safety. Attitude results can guide interventions such as workflow changes to reduce feeding-related stress, simulation to build confidence in aspiration recognition, and reflective practice to help nurses separate their own values from resident-centred decisions. The tool can also be used to evaluate the impact of training programmes and quality improvement projects in long-term care.

This study represents the initial phase of instrument development and exploratory validation for the EATS Questionnaire. The use of EFA was appropriate at this stage to identify the underlying factor structure and refine the attitude items before proceeding to model confirmation. As the EATS was newly developed for nursing home nurses in Singapore, no directly comparable local tools were available to enable meaningful convergent or discriminant validity testing. Future studies will, therefore, include confirmatory factor analysis (CFA) in a separate and larger independent sample to verify the three-factor model, as well as evaluation of convergent validity by comparing EATS scores with related constructs such as empathy, feeding-care confidence, or palliative care orientation. These steps will help to strengthen construct validity and support broader generalisability of the tool across long-term-care settings.

This study has several strengths. The questionnaire was adapted from an earlier instrument and refined through expert and face review to ensure relevance in nursing homes. It was validated in three nursing homes with over 100 participants, giving a representative sample. The final tool combines statistical testing with clinical judgment, ensuring that it is both credible and useful.

Limitations include the modest reliability of the knowledge scale, the single-country validation, and the lack of test–retest or responsiveness testing. The study used a convenience sample from three nursing homes, which may limit generalisability to other facilities or countries. Future research should examine these aspects and explore domain-specific subscales that may provide higher reliability.

## 5. Conclusions

The EATS Questionnaire is a validated tool that captures both essential knowledge and the attitudes that influence dysphagia care among nursing home nurses. The knowledge scale, while modest in reliability, preserves clinically important items that address safety and misconceptions. The attitude scale has a clear three-factor structure with acceptable internal consistency. EATS is useful for identifying knowledge gaps, understanding barriers to care, guiding targeted education, and evaluating the outcomes of training and service improvement. Further research should refine subscales, establish test–retest reliability, and validate the tool in other care settings and cultural contexts.

## Figures and Tables

**Table 1:** Participant Demographics (*N* = 111).

Characteristic	*n* (%)/Mean (SD)
Age (years)	31.4 (6.5)
Length of experience in current occupation (years)	7.3 (10.0)
Length of experience in palliative care (years)	1.8 (3.4)
**Job grade**	
Healthcare Assistant	14 (12.6)
Nursing Aide	52 (46.8)
Senior Nursing Aide	4 (3.6)
Enrolled Nurse	16 (14.4)
Staff Nurse	21 (18.9)
Senior Staff Nurse	2 (1.8)
Director of Nursing	1 (0.9)
Other	1 (0.9)
**Race**	
Filipino	64 (57.7)
Indian	22 (19.8)
Myanmese	13 (11.7)
Malay	2 (1.8)
Chinese	1 (0.9)
Other	9 (8.1)
**Nationality**	
Philippines	63 (56.8)
Myanmar	14 (12.6)
Singaporean	4 (3.6)
Malaysian	1 (0.9)
Other	29 (26.1)

Note: Age ranged from 22 to 53 years. Length of experience in current occupation ranged from 0 to 72 years, and length of experience in palliative care ranged from 0 to 28 years.

**Table 2:** Knowledge and Attitude Scores by Designation.

Designation	Knowledge Mean (SD)	Attitude Mean (SD)	*n*
Healthcare Assistant	14.1 (1.9)	52.1 (10.5)	14
Nursing Aide	15.1 (1.9)	52.6 (6.3)	52
Senior Nursing Aide	15.3 (1.9)	60.0 (9.1)	4
Enrolled Nurse	16.0 (1.5)	50.8 (4.7)	16
Staff Nurse	16.1 (1.6)	52.2 (8.0)	21
Senior Staff Nurse	18.5 (0.7)	51.0 (0.0)	2
Director of Nursing	18.0 (−)	43.0 (−)	1
Overall *p*-value †	0.004	0.35	

† Values are from Kruskal–Wallis tests across designations.

**Table 3:** Correlation of Knowledge and Attitude Scores with Years of Experience.

Predictor	Knowledge (ρ)	*p* Value	Attitude (ρ)	*p* Value
Years of nursing	0.04	0.65	−0.04	0.64
Years in palliative care	0.13	0.16	0.04	0.71

Values are Spearman’s correlation coefficients (ρ), two-tailed.

## Data Availability

The raw data supporting the conclusions of this article will be made available by the authors on request.

## Supplementary Materials

The following supporting information can be downloaded at: https://www.mdpi.com/article/10.3390/nursrep15110405/s1, Table S1: Item analysis and decisions for the Knowledge Scale; Table S2: Rotated Factor Matrix for the Attitude Scale (Varimax rotation).

## Public Involvement Statement

No public involvement in any aspect of this research.

## Guidelines and Standards Statement

This manuscript was drafted against the STROBE (Strengthening the Reporting of Observational Studies in Epidemiology) for cross-sectional validation research.

## Abbreviations

The following abbreviations are used in this manuscript:

EATS	Evaluating Attitudes, Training and Skills
KR-20	Kuder-Richardson 20
EFA	Exploratory Factor Analysis
KMO	Kaiser-Meyer-Olkin
Q	Question
MDQ	Mealtime and Dysphagia Questionnaire
CMDQ	Caregiver Mealtime and Dysphagia Questionnaire
ST	Speech Therapist
NH	Nursing Home

## Appendix A. Final Version After Item Discrimination and Exploratory Factor Analysis

EATS (Evaluating Attitudes, Training and Skills in Dysphagia Care) Questionnaire on Knowledge and Attitudes

This questionnaire measures your knowledge and attitudes towards patients with dysphagia.

**Table A1:** Background Information.

Please fill your job information as requested below.	
Occupation	
Age	
Race	
Nationality	
Length of experience in current occupation	
Length of experience in palliative care	

**Table A2:** Knowledge.

Please answer the questions with True (T)/False (F)/Uncertain (U).		
**Part 1: Swallowing impairment can cause the following problems:**		
1.	Aspiration Pneumonia	T
2.	Social Isolation	T
3.	Giddiness	F
4.	Depression	T
5.	Reduced oral intake	T
6.	Cough	T
7.	Gastritis	F
**Part 2: The following are signs of aspiration:**		
8.	Cough	T
9.	Wet voice	T
10.	Shortness of breath	T
11.	Drop in SpO_2_	T
12.	Drooling	F
13.	Retained food in mouth post swallowing	F
14.	Drop in blood pressure	F
**Part 3: The following questions are about the use of modified diet and fluid:**		
15.	Modified diet requires less effort to chew and manipulate.	T
**Part 4: The following questions are related to patients with dysphagia:**		
16.	Patients with dysphagia should be monitored when being fed.	T
17.	Straw drinking is safer than cup drinking.	F
18.	Spoon drinking is safer than cup drinking.	T
19.	Patients must be checked for food in the mouth after feeding.	T
20.	Good oral care helps to minimize aspiration pneumonia.	T
**Part 5: The following questions are related to enteral/tube feeding (NGT/PEG):**		
21.	Patients on full enteral feeding will not get aspiration pneumonia.	F
22.	Enteral feeding helps prolong the life of terminally ill patients.	F

**Table A3:** Attitudes.

Please answer the following questions using a scale of 0 to 5.				
1	2	3	4	5
Strongly Disagree		Somewhat agree		Strongly agree
**Importance of preventing aspiration**				**0 to 5**
1. It is important to prevent aspiration in my patients.				
2. Patients’ wishes for certain types of food are more important than their risk of aspiration.				
3. Patients should be allowed all diet consistencies since the aim is quality of life rather than to prolong life.				
**Patients’ preference**				**0 to 5**
4. Most of my patients will not want thickened fluids.				
5. Most of my patients will not want pureed diet.				
6. Most of my patients will not want PEG/NGT				
**Perceived barriers to dysphagia care**				**0 to 5**
7. I am confident in looking out for signs of aspiration while feeding my patients.				
8. I find it difficult to prepare thickened fluids for my patients.				
9. It is too time consuming to prepare thickened fluids for my patient.				
10. It is difficult to educate family members on modified diet/fluid.				
11. Modified diet/fluid does not help my patients.				
12. Enteral feeding does not help my patients.				
13. Enteral feeding makes nursing care easier for me.				
14. My patients would not follow the modified diet and fluid recommendations.				
15. I find it stressful to feed patients with dysphagia.				
**Personal choice**				**0 to 5**
16. If I am at risk of aspiration, I would agree to drinking thickened fluids.				
17. If I am at risk of aspiration, I would agree to take pureed diet.				
18. If I am at risk of aspiration, I would agree to NGT/PEG.				
**Any comments or feedback?**				
