# Supplementary material for: Development and Validation of the Evaluating Attitudes, Training and Skills in Dysphagia Care (EATS) Questionnaire Among Nursing Home Nurses in Singapore
Source: Nurs Rep. 2025 Nov 17;15(11):405. doi: 10.3390/nursrep15110405 (PMC12655368; doi:10.3390/nursrep15110405)
Supplement: Supplementary file 1 [file nursrep-15-00405-s001.zip › nursrep-3936701-supplementary.pdf]

Supplementary Materials:

Table S1. Item analysis and decisions for the Knowledge Scale.

Domain	Item	Content (abbreviated)	Difficulty (% correct)	Item-total correlation	Decision	Rationale
**Complications of swallowing impairment**	1	Aspiration pneumonia	97%	.156	Retained	Basic but essential knowledge
	2	Social isolation	53.6%	.416	Retained	Good discrimination
	3	Giddiness	47.3%	.408	Retained	Moderate difficulty, good correlation
	4	Depression	80%	.209	Retained	Clinically relevant
	5	Reduced oral intake	98%	.054	Retained	Core knowledge
	6	Cough (as complication)	98%	.147	Retained	Conceptually important, tests common misconception
	7	Gastritis	19.1%	.269	Retained	Moderately difficult, contributes discrimination
**Signs of dysphagia**	8	Cough	99%	.038	Retained	Widely known but essential
	9	Wet voice	91%	.203	Retained	Good clinical relevance
	10	Shortness of breath	96.4%	.253	Retained	Accepted sign
	11	Drop in SpO_2	95.5%	.297	Retained	Important sign
	12	Drooling	14.5%	.320	Retained	Difficult item, captures misconception
	13	Retained food in mouth	9.1%	.127	Retained	Very difficult, but clinically important
	14	Drop in blood pressure	31.8%	.485	Retained	Strong discrimination
**Modified diets and fluids**	15	Thickened fluids safer	5.5%	.240	**Removed**	Low correlation and misleading
	16	Thickened fluid flows slower	90%	.121	**Removed**	Low correction, limited clinical utility
	17	Modified diet = same caloric content	16.4%	.325	**Removed**	Average correlation, but limited clinical utility
	18	Modified diet easier to chew	82.7%	.250	Retained	Clinically relevant
**Safe feeding practices**	19	Monitoring during feeding	99.1%	.077	Retained	Basic safety
	20	Only trained personnel should feed	78.2%	.053	**Removed**	Low correlation, limited clinical utility
	21	Straw safer than cup	28.2%	.323	Retained	Tests misconception
	22	Spoon safer than cup	97.3%	.112	Retained	Core knowledge
	23	Check for food residue	100%	–	Retained	Clinical standard
	24	Oral care prevents aspiration	95.5%	.052	Retained	Basic but essential
	25	Dependent feeding lowers pneumonia risk	41.8%	.202	**Removed**	Low correlation, not clinically valid
**Enteral feeding**	26	Enteral feeding prevents aspiration	81.8%	.113	Retained	Relevant to misconceptions
	27	Enteral feeding prolongs life	21.8%	.164	Retained	Important palliative care concept
	28	Enteral feeding ↑ aspiration	55.5%	–.026	**Removed**	Negative correlation, confusing

	29	Enteral feeding never appropriate	75.5%	–.049	**Removed**	Negative correlation, confusing

Item analysis of the 30 knowledge items in the EATS questionnaire, showing item difficulty, item–total correlations, and decisions on retention or removal. Items were removed if they demonstrated poor discrimination (item–total correlation <0.20), extreme difficulty indices (≥95% or ≤10% correct), or limited clinical relevance. Items with weaker psychometric properties but judged to be conceptually essential for dysphagia care were retained to preserve content validity.

Table S2. Rotated Factor Matrix for the Attitude Scale (Varimax rotation).

Item (shortened)	Factor 1: Barriers	Factor 2: Patients' Preferences & Nurses' Confidence	Factor 3: Personal Choice
11. Thickened fluids difficult	**0.74**	–0.39	–0.16
12. Time consuming to prepare	**0.75**	–0.25	–0.17
14. Modified diet does not help	**0.76**	–0.28	–0.07
17. Patients won't follow diet	**0.62**	0.25	0.08
15. Enteral feeding does not help	**0.58**	–0.33	–0.35
13. Educating families difficult	**0.48**	0.01	0.07
20. Feeding stressful	**0.51**	–0.15	0.14
16. Enteral feeding makes care easier	**0.50**	0.15	0.38
7. Patients won't want thickened fluids	0.13	**0.72**	–0.05
8. Patients won't want puree diet	0.01	**0.79**	0.02
9. Patients won't want NGT/PEG	–0.09	**0.59**	0.12
10. Confident detecting aspiration	–0.22	**0.55**	0.18
1. Important to prevent aspiration	–0.29	**0.48**	0.24
6. Thickened fluids improve QoL	–0.01	0.21	**0.47**
21. Would take thickened fluids (self)	–0.23	0.09	**0.86**
22. Would take puree diet (self)	–0.15	0.14	**0.88**
23. Would accept NGT/PEG (self)	0.08	–0.09	**0.61**
2. Patients' wishes > aspiration risk	0.39	0.14	–0.17
3. Allow all consistencies (QoL priority)	0.40	0.13	–0.15
4. Preventing aspiration won't improve QoL	**0.52**	–0.24	–0.28
5. Preventing aspiration won't reduce symptoms	**0.49**	–0.24	–0.13
18. Cruel to deny patients food	0.34	0.34	–0.25
19. Patients not worried about aspiration	0.35	0.25	0.03

Rotated factor matrix of the 23 attitude items from the EATS questionnaire using principal axis factoring with varimax rotation. Factor loadings ≥0.40 were considered significant. Items with low loadings (<0.40) or conceptually ambiguous wording (Q4, Q5, Q6, Q18, Q19) were subsequently removed. The final attitude scale comprised 19 items grouped into three factors: (1) Barriers to Dysphagia Care, (2) Patients' Preferences and Nurses' Confidence, and (3) Personal Choice.
